# Editorial: Unravelling complex biobehavioural contributors to obesity: omics, neural, and environmental considerations

**DOI:** 10.3389/fgene.2024.1371642

**Published:** 2024-02-06

**Authors:** Daiva E. Nielsen, Shana Adise, Xue Sun Davis

**Affiliations:** ^1^ School of Human Nutrition, McGill University, Montreal, QC, Canada; ^2^ Division of Endocrinology, Diabetes and Metabolism, Children’s Hospital Los Angeles, Los Angeles, CA, United States; ^3^ Yale School of Medicine, New Haven, CT, United States

**Keywords:** obesity, ingestive behavior, genetics, neuroimaging, life course, childhood circumstances

## Introduction

Obesity rates continue to increase around the world, despite several individual and population-based efforts that target weight management (Branca et al., 2023). While the principle of energy homeostasis has been a primary framework for obesity, the diverse biobehavioral considerations connected to energy intake require further attention (Vainik et al., 2018; Hall et al., 2022). These include omics, neural, and environmental considerations, and their potential interrelationships or interactions, which contribute to obesity-related traits. This Research Topic aimed to highlight novel research on complex biobehavioral contributors to obesity. The articles in this Research Topic have illuminated intriguing areas for continued investigation, including the broad themes summarized below.

## Early life diet, brain morphology, and eating behaviors

Exposures in the first 1,000 days of life, including life *in utero*, are known to be a critical period of development (Karakochuk et al., 2018). Breastfeeding is related to numerous benefits in early life, childhood, and beyond, but little is known about the links between breastfeeding and neurodevelopment. Understanding these links are critical because early life nutrition may shape appetitive drives. Using data from the COGNIS study, Dieguez et al. assessed patterns of associations between infant feeding practices in the first 18 months of life (breastfed, standard formula, or experimental bioactive-enriched formula groups) and hypothalamic functional connectivity (FC) at age 6 years, assessed with functional magnetic resonance imaging (fMRI) (Dieguez et al.). Differences in FC were observed in brain regions that are involved in eating motivation and hedonic eating behaviors between all three infant feeding practice groups. The most pronounced differences were reported between children who were breastfed compared to those fed with standard formula during infancy. Moreover, at 6 years old, children who were breastfed as infants had lower blood glucose levels compared to those who were fed with standard formula, suggesting that infant diet may be related to later childhood eating behaviors or metabolic traits, potentially through influences on neurobiological development.

Pearce et al. also investigated the neurobiology of eating behavior, by examining whether there were neurobiological differences in brain structure associated with food intake and loss of control eating trait (LOC) in childhood (Pearce et al.). LOC is the perceived inability to control how much is eaten, regardless of actual amount consumed, and is a risk factor for development of Binge Eating Disorder (BED). Using structural MRI neuroimaging data the authors reported that children with LOC (compared to those without, mean age 8.9 years) had structural differences in brain regions related to cognitive control, reward-related decision-making, and regulation of eating behaviors. This work suggests that alterations in the brain appetite regions may unravel precursors of BED amongst children exhibiting LOC.

In addition to neurobiological considerations, the role of genetics in eating behavior is being increasingly investigated (Silventoinen and Konttinen, 2020). Jansen et al. examined links between child genetic obesity risk and parental feeding practices using data from the RESONANCE study (child mean age 7.5 years) (Jansen et al.). The authors reported that two parental feeding practices (restriction for weight control and teaching about nutrition) were significantly associated with child polygenic risk score for body mass index (PRS BMI), which may exert effects on body weight through appetitive traits. These associations were independent of child BMI z-score. Moreover, compared to children with parent-reported low food responsiveness, parents were more likely to restrict child food intake for weight control purposes for children with a high PRS BMI who showed moderate/high food responsiveness. These observations suggest that amongst children who are genetically at risk for obesity, parents of children who are high food responders may inadvertently adjust their feeding practices in an attempt to control the child’s weight.

## Functional studies of obesity genes and interactions with environmental conditions

While the above studies utilized child populations to study complex contributors to obesity, animal studies provide valuable insight into the mechanisms that may be driving these associations. Two articles in this Research Topic report findings from rat studies that propose new mechanisms into possible gene-environment interactions and obesity risk. Szalanczy et al. examined the effects of the Keratinocyte-associated protein 3 (*Krtcap3a*) gene on adiposity traits by developing a whole-body *Krtcap3a* knock-out (KO) rat model. While the physiological function of *Krtcap3a* remains to be elucidated, it has been previously identified as a candidate gene for adiposity (Keele et al., 2018). In their first study, wild-type (WT) and KO rats were placed on a high-fat or low-fat diet for 13 weeks (Szalanczy et al.). Compared to WT, KO rats had significantly greater weight gain, particularly among high-fat diet KO females. KO females also ate more and had greater adiposity than WT rats. In their second study, the authors incorporated consideration of environmental stress into assessments of *Krtcap3* and adiposity traits (Szalanczy et al.). When exposed to stress, KO rats consumed more food and gained more weight compared to KO rats unexposed to stress. Moreover, minimal differences were observed in adiposity-related outcomes between WT rats regardless of stress exposure condition (exposed vs. unexposed). Together, these findings suggest that *Krtcap3* plays a role in obesity-related traits, with potential sex-specific effects and mechanisms that interact with stress response.

## Conclusion and future directions

The articles in this Research Topic showcase that obesity risk is partly shaped by diverse complex biobehavioral factors that interact with environmental conditions potentially from the start of life. Knowledge gaps remain in understanding how genetic variation relates to the neurobiology of eating behaviors and how diverse environmental conditions, such as diet and stress, may interact with genetic variation on eating behavior. Future research, particularly longitudinal integrative studies applying multi-omics, neuroimaging, and systems biology, will further illuminate the life course relationships between biology and behavior that contribute to obesity risk and can lead to development of innovative, more effective approaches for prevention and management.
